# Whole Body Cryostimulation: A New Adjuvant Treatment in Central Sensitization Syndromes? An Expert Opinion

**DOI:** 10.3390/healthcare12050546

**Published:** 2024-02-25

**Authors:** Angelo Alito, Federica Verme, Gian Paolo Mercati, Paolo Piterà, Jacopo Maria Fontana, Paolo Capodaglio

**Affiliations:** 1 Department of Biomedical, Dental Sciences and Morphological and Functional Images, University of Messina, 98125 Messina, Italy; alitoa@unime.it; 2 Research Laboratory in Biomechanics, Rehabilitation and Ergonomics, IRCCS, Istituto Auxologico Italiano, San Giuseppe Hospital, Piancavallo, 28824 Verbania, Italy; f.verme@auxologico.it (F.V.); p.pitera@auxologico.it (P.P.); p.capodaglio@auxologico.it (P.C.); 3 Department of Surgical Sciences, Degree Course in Physiotherapy, University of Torino, 10121 Torino, Italy; ps2976@edu.unito.it; 4 Department of Surgical Sciences, Physical and Rehabilitation Medicine, University of Torino, 10121 Torino, Italy

**Keywords:** central sensitization, chronic pain, cryotherapy, whole-body cryostimulation, widespread pain

## Abstract

Central sensitisation is defined as a multifactorial etiopathogenetic condition involving an increase in the reactivity of nociceptive neurons and alterations in pain transmission and perception in the central nervous system. Patients may present with widespread chronic pain, fatigue, sleep disturbance, dizziness, psychological (e.g., depression, anxiety, and anger) and social impairment. Pain can be spontaneous in onset and persistence, characterised by an exaggerated response and spread beyond the site of origin, and sometimes triggered by a non-painful stimulus. Whole-body cryostimulation (WBC) could be an adjuvant therapy in the management of this type of pain because of its global anti-inflammatory effect, changes in cytokines and hormone secretion, reduction in nerve conduction velocity, autonomic modulation, and release of neurotransmitters involved in the pain pathway. In several conditions (e.g., fibromyalgia, rheumatoid arthritis, and chronic musculoskeletal pain), WBC affects physical performance, pain perception, and psychological aspects. Given its multiple targets and effects at different organs and levels, WBC appears to be a versatile adjuvant treatment for a wide range of conditions of rehabilitation interest. Further research is needed to fully understand the mechanisms of analgesic effect and potential actions on pain pathways, as well as to study long-term effects and potential uses in other chronic pain conditions.

## 1. Introduction

Central sensitization (CS) is a multifactorial etiopathogenetic condition involving an increase in the reactivity of nociceptive neurons and alterations in pain transmission and perception in the central nervous system [1,2]. It results in hyperexcitability to noxious and non-noxious stimuli that act on structural plasticity, and may sensitise the central nociceptive system [3,4]. When CS occurs, neurons in the spinal cord become more responsive to peripheral input, such as below-threshold stimuli that would not normally evoke a pain response [4,5]. This augmented sensitivity can also result in an expansion of the receptive field of the dorsal horn neurons, enhancing the transmission of nociceptive information to the somatosensory cortex [6,7]. Patients may also report global hypersensitivity to external stimuli, such as intense light, noise, odours, certain foods, or drugs [8,9].

These characteristics define so-called Central Sensitization Syndromes (CSS), in which chronic widespread pain is the central feature and other overlapping disorders, such as fatigue, sleep disturbances, dizziness, psychological (e.g., depression, anxiety, and anger) and social (e.g., interpersonal distress) impairments may coexist [10,11].

**Figure 1:**
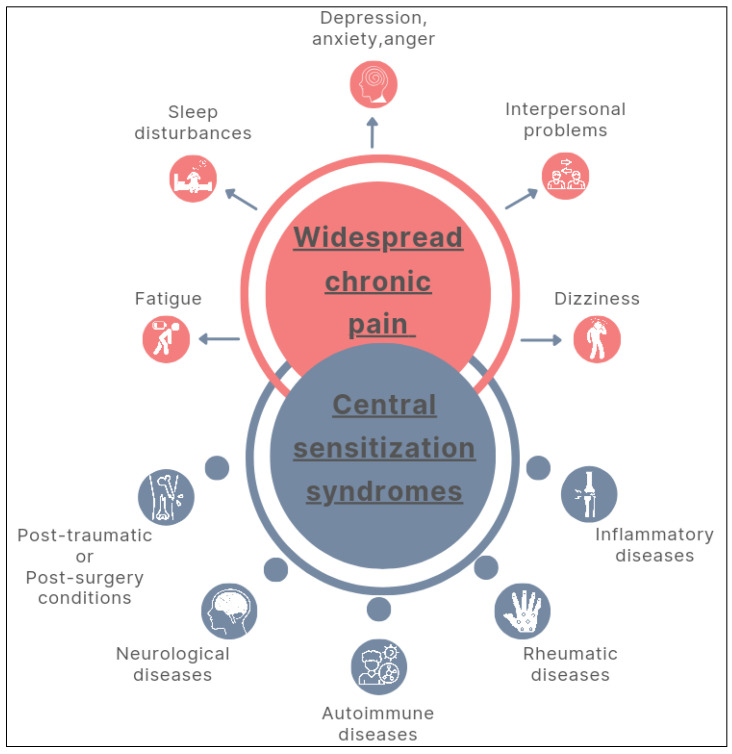
Multidimensional nature and overlapping manifestations of central sensitization syndromes.

CSS encompasses inflammatory, rheumatic, autoimmune, neurological, post-traumatic, or post-surgical conditions (Figure 1) [12,13,14]. The aetiology of CSS is not fully known yet, and may be induced by the combination or overlap of autoimmune, genetic, and environmental factors, as well as changes in the sympathetic nervous system [4]. These include peripheral and central sensitisation, inflammation, and altered sympathetic function [11].

As a result, pain resulting from these mechanisms may have the following characteristics: (i) spontaneous onset and persistence, even in the absence of a stimulus; (ii) triggered by non-painful stimuli (allodynia); (iii) excessive response, both in intensity and duration, to painful stimuli (hyperalgesia); and (iv) spread beyond the site of origin (secondary hyperalgesia) [4].

This pain is defined as nociplastic pain, which results from changes in the processing of pain signals and dysfunction within the central nervous system in the absence of peripheral nociception or tissue damage [15].

The role of environmental and psychological factors in the development and exacerbation of CS symptoms is widely recognized, as are altered perception of reality, catastrophism, and kinesiophobia [16,17].

The persistence of symptoms may lead to limitations in daily activities, discomfort, loss of functional significance of pain alertness, and development of a range of associated symptoms, such as increased sensitivity to tactile, auditory, and visual stimuli [18,19]. Given the multiple etiopathogenetic mechanisms and the range of possible manifestations, a multimodal and customised clinical approach to CSS is needed [20,21]. In fact, when CS pain is present, a biomedical approach that focuses on the underlying pathology or peripheral symptoms is ineffective in most cases [20].

The presence of CS is correlated with worse long-term rehabilitation outcomes, increased drug consumption, and longer recovery [22]. Its management consists of ceasing or reducing the neuronal hyperactivity through a combination of pharmacological and non-pharmacological treatments together with the treatment of associated symptoms, as these contribute to worsening pain and disability [1].

Pharmacological treatments attempt to modulate neurotransmitter activity or target specific receptors involved in pain processing. In particular, tricyclic antidepressants and selective serotonin-norepinephrine reuptake inhibitors have shown efficacy in reducing neuronal hyperexcitability, while anticonvulsants such as gabapentin and pregabalin are commonly prescribed to reduce the release of excitatory neurotransmitters [20].

Non-pharmacological treatments are based on psychological approaches that aim to modify the dysfunctional thoughts, emotions, and behaviours associated with pain, and to change the brain representation of affected body parts; on regular physical activity and exercise to modulate pain processing, improve function, and reduce disability; on manual therapy to reduce muscle tension, improve joint mobility, and relieve pain; and on education and self-management strategies that can empower individuals to take an active role in managing their symptoms [22,23].

The complex interaction between the sympathetic nervous system and the perception of pain in CSS highlights the need to explore novel therapeutic approaches; attempts to desensitise the central nervous system may be unsuccessful and adjunctive treatments (cognitive-behavioural-educational therapies, therapeutic exercise, stress management techniques) are often used [24,25,26].

Cryotherapy has gained attention in the field of pain management for its potential to reduce discomfort and improve overall well-being [27]. The effects are the reduction of inflammation, induction of vasoconstriction resulting in decreased blood flow to the affected area, numbing of nerve endings with reduced transmission of pain signals, and modulation of the release of neurotransmitters, promoting relaxation of muscle fibres leading to pain relief and improved mobility [28].

There are various methods of exposing the body to low temperatures, such as immersion in cold water, ice packs or cooling devices, which can produce a variable temperature distribution depending on the part of the body affected, the cooling method, and the duration of exposure [29].

Whole-body cryostimulation (WBC) is a physical treatment based on brief (2–3 min), repeated exposures of the whole body to extremely low temperatures (−110° to −140 °C) in special cryogenic chambers. It has shown rapid anti-inflammatory and antioxidant effects, pain, and autonomic modulation capacity, and for those reasons it has been used as an adjuvant therapy in several conditions [30,31,32]. Safety of WBC and possible adverse events have recently been reviewed: when accurate medical screening is performed, WBC is a safe, well-tolerated treatment, and reported adverse events are rare [33,34].

As clinical studies on WBC are taking off, we felt that discussing the possible mechanisms of action of WBC on the central nervous system based on the data available from the literature and on the authors’ expert opinion and personal experience at San Giuseppe Hospital (IRCCS Istituto Auxologico, Piancavallo, Italy) was timely and of interest to rehabilitation specialists.

## 2. Biology and Immunology of CS

**Figure 2:**
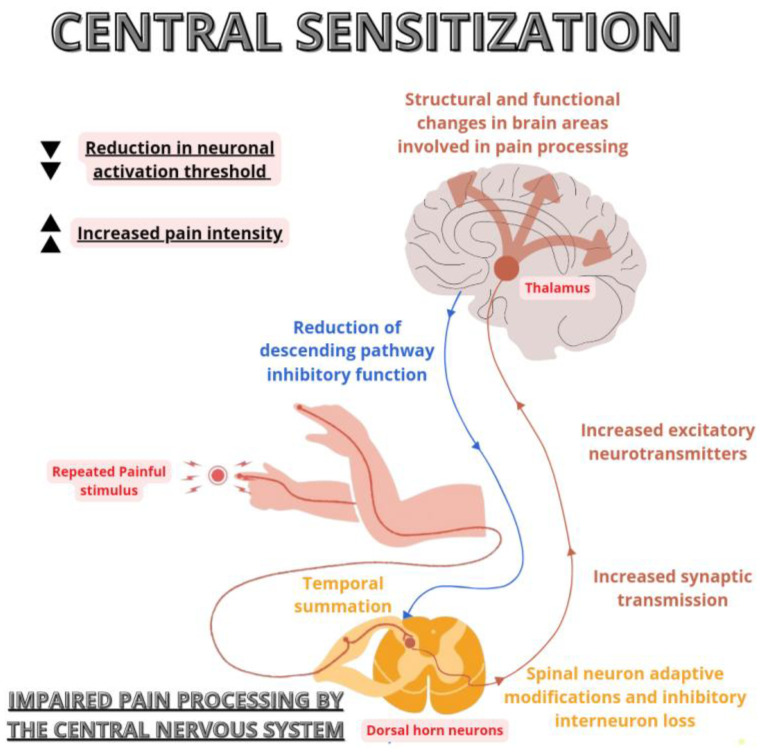
A schematic illustration of the neurobiological processes underlying central sensitisation, showing the increased responsiveness of the central nervous system to nociceptive stimuli.

At the biological level, CS is characterised by an increase in excitatory neurotransmitters and synaptic efficacy, a reduction in the inhibitory function of descending pain pathways, a loss of inhibitory interneurons, and an alteration of supraspinal processes responsible for pain perception and in brain regions involved in emotional responses (Figure 2) [3,35].

Among the mechanisms responsible for CS, temporal summation plays a key role, and the presence of a prolonged nociceptive afferent stimulus induces an increase in neuronal excitability in nociceptive pathways, leading to a reduction of the neuronal activation threshold and an increase in pain intensity [35].

After prolonged stimulation of nociceptive fibres, glutamate may be released for longer than usual, leading to activation of normally inactive N-methyl-D-aspartate (NMDA) receptors and an increase in intracellular calcium in the spinal cord postsynaptic neurons, making these second-order neurons more responsive to stimuli [36]. Prolonged maintenance of this process, at both pre- and post-synaptic levels, produces adaptive changes in the spinal nerve cell, resulting in the altered perceptual phenomena typical of CS, becoming chronic in advanced stages [2].

Functional imaging in patients with chronic pain has shown structural and functional changes in brain areas involved in pain processing with expansion and reorganisation of the thalamus, insula, periaqueductal grey, and somatosensory cortex [37].

In addition, an increase in excitatory (glutamate and substance P) and a decrease in inhibitory transmitters (i.e., gamma-aminobutyric acid, GABA) occur together with a downregulation in dopaminergic activity and opioid receptors [4].

Changes in A-beta nerve fibres, physiologically responsible for transmitting non-noxious impulses associated with tactile stimulation to the spinal cord, also contribute to increased pain transmission [35]. These fibres tend to colonise the posterior horns of the spinal cord in the presence of sensitization and synapse with second-order nociceptive neurons leading to the activation of pain pathways even in response to low-threshold non-noxious stimuli [6].

The immune system also plays a role in the development of CS, responding to stressors by increasing pro-inflammatory cytokines, particularly interleukin IL-1, IL-6, and tumour necrosis factor α (TNF-α) in the spinal cord [38]. The presence of these proinflammatory molecules increases excitatory synaptic transmission, enhancing the function of excitatory α-amino-3-hydroxy-5-methyl-4-isoxazolepropionic acid (AMPA) and NMDA and suppressing inhibitory synaptic transmission mediated by GABA and glycine [39]. Cytokines are thus directly responsible for enhancing pain transmission and influencing sensitization-related symptoms such as hyperalgesia, allodynia, fatigue, cognitive dysfunction, stress, and anxiety [29]. CSS are also often associated with dysregulation of the hypothalamic-pituitary-adrenal system, resulting in decreased cortisol production and subsequent hyper- or hypo-cortisolism, which alters the pain signal and sometimes sensitises nearby nociceptive afferents [40]. As a result of the above changes, reduced central nervous system function is observed with excitatory activity predominating over inhibitory activity [37].

## 3. Effects of WBC on Pain

**Figure 3:**
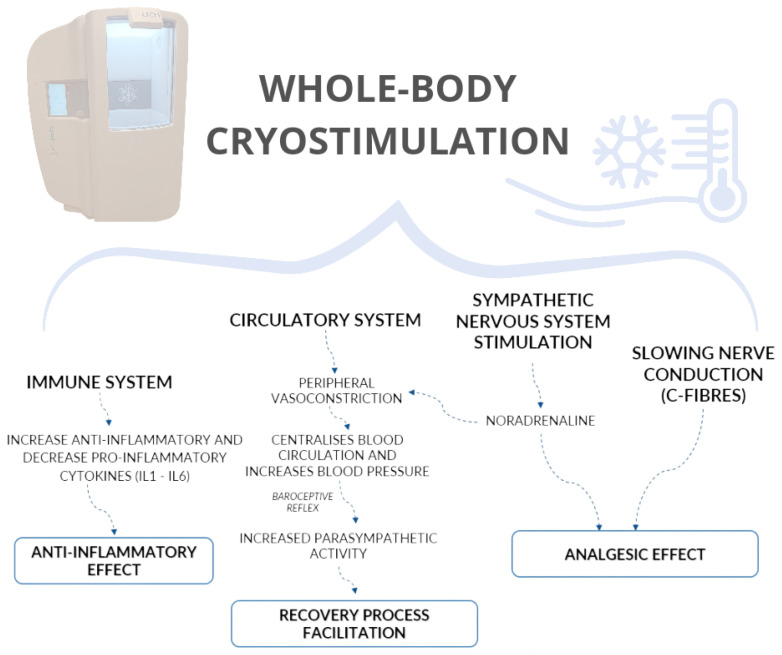
Overview of the impact of whole body cryostimulation. A diagram detailing its multiple and systemic benefits, including immune modulation, anti-inflammatory responses, and circulatory effects. A graphic roadmap for understanding the wide-ranging implications of this emerging therapeutic approach.

Exposing the human body to cold activates two mechanisms to maintain core temperature and reduce heat loss: vasomotor responses, aimed at reducing dry heat loss to the environment, and metabolic responses, which act to replace heat lost to the environment through heat production, or thermogenesis [41]. Moreover, the musculoskeletal, cardiovascular, respiratory, nervous, and endocrine systems may be affected by WBC via neuroendocrinological, humoral, and immune regulatory mechanisms [31]. Cold temperatures enhance the body’s natural anti-inflammatory responses, modulate neurotransmitters involved in pain signalling, and increase the release of analgesic factors such as serum beta-endorphin and norepinephrine [42,43]. WBC also act on the hypothalamic thermoregulatory centre, causing activation of the sympathetic system, which modulates the pain response by acting on nerve conduction (i.e., C-fibres), inhibiting sensory receptors and their connections to proprioceptors [44,45] and releasing brain beta-endorphins [46]. Cold stress stimulates the thermal receptors in the dermis by reducing skin temperature, causing vasoconstriction, and slowing the conduction of pain nerves, which has an analgesic effect [44,46]. Figure 3 provides a comprehensive overview of the effects of WBC.

As a result, WBC has been shown to reduce inflammation, which can contribute to peripheral sensitisation, and inhibit nociceptive transmission in the spinal cord, thus reducing CS and nociplastic pain [47].

There are also effects on the endocrine system and hormone levels, but findings are not entirely convincing. In fact, some studies suggest that WBC may transiently increase testosterone levels in response to cold stress, considering its effects on the regulation of metabolism, energy expenditure, and thermoregulation, but results are inconsistent on significant long-term effects [48]. In addition, an initial increase in cortisol levels can be found, which may decrease over time as part of an adaptation phenomenon [49]. However, further research is needed to fully understand the effects of WBC on stress hormone levels, especially in patients with CSS.

WBC may also trigger psychological responses, due to an increased sensation of well-being secondary to strengthening of parasympathetic tone and the release of endorphins, ultimately leading to a reduction in pain perception and CS [50].

## 4. WBC in CSS: Evidence from the Literature

As previous research has shown, CS and WBC may have a significant interaction on the effect on disease impact, quality of life, and pain perception, suggesting its potential in modulating sensitisation and reducing pain [51].

Therefore, understanding the specific pathways and physiological changes induced by WBC in relation to CS may provide valuable insights for the development of novel pain management strategies and interventions.

As indicated in a recent review, WBC may be an adjuvant therapy in the reduction of chronic pain as a result of lower inflammation, oxidative stress, and nerve transmission velocity in pain fibres [28].

This is a key concept in rehabilitation, as reducing fatigue and pain, improving range of motion, and facilitating recovery could enhance adherence to the rehabilitation program, improve mood, and lead to better outcomes.

Some CSS treated with WBC as a complementary or alternative therapy to conventional treatment will be reviewed here.

### 4.1. Fibromyalgia

Patients affected by fibromyalgia (FM) show persistent impairment of pain modulation systems, such as central nociceptive hyperexcitability or enhancement, and reduced inhibitory responsiveness [52]. This prolonged impairment, combined with an imbalance between inflammatory and anti-inflammatory cytokines (e.g., IL-1, IL-6, IL-10, and TNF-α), leads to CS and the induction and maintenance of pain perception, sustaining FM-related hyperalgesia [53,54,55]. In FM, WBC may help relieve pain by modulating the release of neurotransmitters and hormones involved in the pain pathway, such as endorphins, serotonin, and norepinephrine, addressing both neurological and psychological components [16,55].

Some studies have reported the positive effects of WBC in FM. Bettoni et al., in their clinical trial involving 100 patients, showed benefits after 10 WBC sessions in terms of pain perception and quality of life compared to the control group (no WBC); similar results were found by Rivera and colleagues studying 60 patients in a randomised, open-label crossover trial (WBC—no WBC group) at a 1-month follow-up [52,56].

In their study, Varallo et al. analysed the effect of a multidisciplinary rehabilitation program, including 10 WBC sessions at −110 °C in a population of 43 patients affected by obesity and FM [31]. They found that the group receiving the WBC intervention had greater improvements in pain severity, depressive symptoms, and disease impact on quality of life and sleep quality than the control group receiving a multidisciplinary rehabilitation intervention [31].

### 4.2. Rheumatological Diseases

In patients affected by Rheumatoid Arthritis, several studies have been conducted using WBC, with inconsistent results. Klemm and colleagues in their single-blinded, randomised controlled trial of 56 patients found a reduction in pain perception during a 16-day multimodal conventional treatment including WBC (six 3 min sessions), with a reduction in pain and serum cytokine levels (TNF and IL-6), compared to the control group who received no treatment [57]. A reduction in these mediators may lead to reduced depolarisation of peripheral nociceptors because of diminished input from ascending neurons in the cortical pain matrix, thereby facilitating a subsequent reduction in pain sensation [58].

In their randomised single-blinded controlled trial, Hirvonen et al. showed that 20 out 60 patients treated with WBC at −110 °C, as an adjunct to physiotherapy, appeared to reduce pain more than other cryotherapies (n = 40), but with a high cost–benefit ratio, and no significant differences in disease activity between groups and with local cryotherapy. However, the study groups were heterogeneous and small [59]. On the other hand, some studies do not show clear evidence of superiority, as Gizinska showed in a trial of 44 patients divided into a WBC group (n = 25) and a traditional rehabilitation group (n = 19), resulting in similar significant reductions in IL-6, TNF-α levels, pain, and physical function [60].

A similar trend was reported by Verme et al. in a case-report on a 74-year-old woman with polymyalgia rheumatica and a two-year history of widespread pain that defined a CS condition [32]. She underwent a 10-session cycle of WBC as a stand-alone treatment, with long-lasting (6 months) positive effects on disease impact, fatigue, pain, sleep quality, and total physical activity, and a concomitant reduction in pharmacological treatment [32].

### 4.3. Chronic Low Back Pain

Patients suffering from low back pain (LBP) often complain of a lack of relief from their symptoms due to ineffective treatments or no treatment at all, and repeated nociceptive stimuli can sometimes lead to chronicity and occasionally to CS [61]. In 2022, Barłowska-Trybulec et al. analysed a group of 30 patients with LBP and found an improvement in pain after a WBC cycle combined with exercise, suggesting an effect on the nervous and endocrine systems in response to cold and stress stimuli, but there was no control group [46]. Similarly, Salas-Fraire et al., in a quasi-experimental study without a control group, involving 41 patients, found that, for chronic LBP, WBC was effective on pain perception and disability after only four sessions, as well as reducing serum markers of inflammation and increasing anti-inflammatory markers [62].

### 4.4. Other Chronic Pain Syndromes

CS may play a role in myofascial pain syndrome, and WBC has been investigated as a possible therapeutic approach, with findings of reduced perceived pain and an increase in pain threshold shortly after WBC [63].

Phantom limb pain (PLP) can also be included in CSS because of the complex interaction between the peripheral and central nervous systems [64,65]. A recent study on an amputated paralympic athlete with phantom limb syndrome and chronic pain due to CS showed a clinically significant reduction in PLP intensity and paraesthesia that lasted for two weeks after WBC and returned at a lower intensity after one month, allowing a better sleep quality than before WBC [66].

Overall, the evidence suggests that WBC has the potential to counteract CS by relieving chronic pain, reducing pain perception, and improving overall well-being.

Breaking the pain cycle can be achieved through interventions that have been shown to effectively reduce pain sensitivity and alleviate symptoms, thereby avoiding or reducing the symptoms of CS [10].

In addition to the physical benefits, there are psychological effects of WBC that may contribute to pain management, including improved mood and reduced fatigue, which may lead to better outcomes in terms of adherence to treatment, and, consequently, improved functional ability, with a potential positive impact on the quality of life of people with CSS.

These effects are already known in the healthy population, such as the recovery of athletes by reducing muscle soreness and inflammation after exercise, accelerating muscle tissue repair, and improving overall performance by promoting better sleep, reducing fatigue, and increasing well-being, contributing to their competitive advantage [67].

Given the multiple targets and effects at different organs and levels, WBC appears a versatile adjuvant treatment across a wide range of conditions of rehabilitation interest, although not always accessible to all patients, and with a relatively high cost–benefit ratio.

WBC could provide a safe and feasible alternative to other cooling methods, such as cold-water immersion, in terms of standardised and personalised exposure conditions for temperature and timing [68].

Although some of the mechanisms of action remain not fully unveiled, the promising, although not yet consolidated, data available in the literature have prompted authors to express their expert position about a possible role of WBC in CS to facilitate further larger studies. In this type of study, it is also important to consider the placebo effect, which may vary depending on several factors, including study design, participants’ expectations of treatment, and the nature of the control group, which may include a sham treatment.

Complementary therapies that promise to achieve rehabilitation outcomes in a shorter time frame should be welcomed in clinical practice and validated in larger populations, given the time and resource constraints physicians often experience in rehabilitation settings.

However, some of the studies analysed presented some limitations. A limitation of some of the studies is the small population size used for data collection. The limited sample may not adequately represent the diverse range of individuals found within the general population, thereby limiting the generalisability of the findings. Therefore, caution should be exercised in extrapolating the conclusions to a wider context, as the characteristics of the sample may not accurately reflect those of the general population.

Another limitation of this study is the inherent challenge of conducting a sham treatment. Due to the nature of the intervention, it may be impractical to create a credible placebo or sham procedure. This limitation may introduce potential bias, as participants and researchers may be aware of the treatment condition, which may influence their behaviour on interpretation of the results.

Moreover, the beneficial effect of WBC could be confounded by the concomitant rehabilitation program or the additional psychological benefit of performing an innovative technique.

Further research is needed to fully understand the mechanisms by which WBC exerts its analgesic effects, and its potential to counteract CS and to investigate the long-term effects and potential application in other chronic pain conditions.

## 5. Conclusions

There is initial evidence that WBC may be an effective treatment for chronic pain and CS. The global anti-inflammatory effect, changes in cytokines and hormone secretion, reduction in nerve conduction velocity, autonomic modulation, release of neurotransmitters and hormones involved in the pain pathway (such as endorphins, serotonin, and noradrenaline), global antioxidant status, and improved immune system function may all together explain the reduction in perceived pain. The nociceptive, neuropathic, central, and psychological components of pain all seem to be addressed by WBC. The exercise-mimicking effects of WBC make cold exposure a possible and feasible alternative intervention to exercise for patients suffering from pain, inflammation, and functional impairments, which may trigger patients’ positive emotions and boost adherence to rehabilitation prescriptions. Finally, it seems necessary to standardise the parameters of time and temperature of exposure in different conditions and populations and to compare the effects with other non-pharmacological interventions for pain management.
